# Have you done your research? Information sources and trust as predictors of vaccine intentions and uptake during Australia’s COVID-19 vaccination rollout

**DOI:** 10.1080/00049530.2026.2716072

**Published:** 2026-09-14

**Authors:** Karina T. Rune, Samuel J. Devonald, Jacob J. Keech

**Affiliations:** a School of Health, University of the Sunshine Coast, Sippy Downs, Australia; b School of Applied Psychology, Griffith University, Brisbane, Australia

**Keywords:** COVID‑19 vaccination, vaccine hesitancy, vaccination misinformation, information sources, trust

## Abstract

**Background:**

The COVID-19 pandemic generated a global “infodemic” of misinformation, shaping public perceptions, trust, and vaccine hesitancy. Although COVID-19 vaccination campaigns have concluded, understanding how people used and trusted information sources during the rollout remains relevant to improving future public health communication.

**Objective:**

This study examined (1) associations between information sources and COVID-19 vaccination intentions and uptake in Australia, (2) the role of trust in these sources, and (3) discrepancies between source use and trust.

**Method:**

A longitudinal survey (*N* = 405; 63.5% female) was conducted at two time points: Time 1, before COVID-19 vaccinations were freely available and Time 2, approximately 7 months later, after vaccines became accessible to all adults.

**Results:**

Trust-based predictors explained more variance than source-use predictors, and ABC news outlets and state government sources emerged as the most consistent positive predictors of both vaccination intention and uptake. In contrast, social media use was negatively associated with both intention and uptake, although trust in social media was not a significant predictor, suggesting that reliance on social media, rather than trust in it, may be most relevant. Participants frequently relied on social media despite reporting lower trust in it.

**Conclusion:**

Findings highlight persistent challenges posed by misinformation and emphasise the need to strengthen trust in credible information sources to support effective public health communication during public health crises.

## KEY POINTS

What is already known about this topic:

1. Misinformation during the COVID-19 pandemic contributed to vaccine hesitancy and lower vaccination uptake.
2. Trust in health professionals, governments, and scientific institutions is central to vaccination decision-making.
3. Social media often amplifies misinformation, contributing to reduced vaccine confidence.

What this topic adds:

1. Using longitudinal data collected before and after the availability of COVID-19 vaccines, this study shows that information sources and trust prospectively predict subsequent vaccination uptake, not just intentions.
2. Trust in health professionals and government sources was a stronger predictor of vaccination behaviour than information use alone, highlighting trust as a key intervention target.
3. This study identifies a systematic gap between trust and information use, demonstrating that individuals rely on less trusted sources, such as social media, even when they recognise these sources as less credible.

## Introduction

Since the onset of the COVID-19 pandemic in early 2020, an unprecedented volume of health information has been disseminated globally through mainstream and social media platforms. Alongside accurate and timely public health communication, there was a rapid proliferation of misinformation and disinformation (Cinelli et al., 2020; Kouzy et al., 2020). The World Health Organization (World Health Organization, 2020) labelled this phenomenon an *infodemic*, highlighting the difficulty of managing information quality and public understanding during large-scale health emergencies. Misinformation refers to false or inaccurate information that is unintentionally spread, whereas disinformation involves the deliberate spread of false information intended to mislead (Dupuis et al., 2021). Both forms of information have been linked to vaccine hesitancy (Lee et al., 2022; Loomba et al., 2021), despite strong scientific consensus regarding the safety and efficacy of COVID-19 vaccines (Jarrett et al., 2015; Sallam, 2021; Wilson & Wiysonge, 2020). Vaccine hesitancy, defined as the reluctance or refusal to vaccinate despite the availability of vaccines, is recognised as one of the top ten global threats to public health (World Health Organization, 2019). This paper examined how different information sources, and the level of trust placed in them, predicted COVID-19 vaccination intention and uptake in Australia during the pandemic.

The COVID-19 infodemic fostered a complex media environment where accurate information was often interspersed with misleading narratives, undermining public health efforts and eroding trust in vaccines (Islam et al., 2020). Misinformation and disinformation contributed to increased vaccine hesitancy, particularly when the information appeared scientifically credible (Lee et al., 2022; Loomba et al., 2021; Raza et al., 2023). In Australia, belief in COVID-19 misinformation was linked to reduced perception of the virus’s threat and decreased trust in government and scientific institutions (Pickles et al., 2021), both key factors associated with vaccine hesitancy. These dynamics reflected broader challenges in maintaining public trust amid scientific uncertainty, evolving evidence, and rapidly changing policy guidance. Inconsistent government messaging and media misrepresentation compounded these challenges, particularly among young Australians influenced by social media (Muhammed & Mathew, 2022; Sum et al., 2024). Although the acute phase of the pandemic has passed, the mechanisms by which misinformation spreads and trust shapes health decision-making remain salient for current and future health crises.

Different information sources, ranging from mainstream media and social media platforms to government communications, varied significantly in their influence on COVID-19 vaccination attitudes and behaviours (Betsch et al., 2020; Thaker et al., 2023). For example, Liu et al. (2023) found that exposure to COVID-19 information from traditional media and online news sources reduced vaccine hesitancy, while exposure to social media increased hesitancy due to the prevalence of misinformation. Likewise, Loomba et al. (2021) demonstrated that online misinformation reduced vaccination intent, emphasising the importance of information channels. In Australia, Thaker et al. (2023) reported that traditional media and health experts influenced some segments of the population, while others were more swayed by social media and personal networks. These findings highlight the need for tailored public health communication strategies to address misinformation and disinformation and promote reliable information sources during large-scale health campaigns.

Trust in information sources also plays an important role in shaping health behaviours, including vaccination. Trust in governmental and scientific institutions has been linked to higher vaccine acceptance and engagement in recommended health behaviours (Jennings et al., 2021; Larson et al., 2014; Loomba et al., 2021). Conversely, low trust has been associated with increased rates of vaccine hesitancy and refusal (Allington et al., 2023; Brewer et al., 2017; Jennings et al., 2021). In Australia, trust in mainstream media and health experts predicted vaccine acceptance during COVID-19 (Choi et al., 2023; Thaker et al., 2023), whereas exposure to social media misinformation reduced vaccine confidence (Choi et al., 2023; Liu et al., 2023; Williams et al., 2024). Low trust was particularly problematic when individuals relied on less-regulated social media sources, which amplified misinformation and limited critical evaluation (Liu et al., 2023).

Conceptually, the present study is informed by complementary perspectives on health behaviour, trust, and media use. The Theory of Planned Behaviour (Ajzen, 1991, 2006) highlights the central role of intentions in shaping behaviour, providing a rationale for examining both vaccination intentions and subsequent uptake. However, intentions are also shaped by broader informational and social contexts, including trust in institutions and exposure to different information sources (Jennings et al., 2021; Larson et al., 2014). Research on media and communication further demonstrates that people’s reliance on information sources is influenced not only by perceived credibility, but also by the accessibility of information and patterns of exposure shaped by contemporary information environments (Betsch et al., 2020; Cinelli et al., 2020). Accordingly, the present study examined the use of information sources and trust as relevant predictors of both vaccination intention and subsequent uptake, consistent with these complementary perspectives.

The research described above highlights the role of information sources and trust in information in determining vaccination-related behaviour. While Australian studies have examined misinformation, trust, or media influences on COVID-19 vaccine attitudes (e.g., Pickles et al., 2021; Thaker et al., 2023), these factors have not been examined together, using a prospective longitudinal design that captures both vaccination intentions and subsequent uptake, nor has prior Australian work examined systematic discrepancies between information source use and trust across a broad media environment.

The current study was conducted during a critical period of the COVID-19 vaccine rollout in Australia, providing a unique opportunity to examine information use and trust in a large-scale public health emergency, with relevance to COVID-19, future health crises, and routine immunisation contexts. Accordingly, the study had three aims. First, we examined associations between COVID-19 vaccine information sources and vaccination intentions and uptake. Second, we examined whether trust in these information sources was associated with vaccination intentions and uptake. Third, we sought to investigate any discrepancies between COVID-19 information sources and the level of trust placed in them.

## Methods

### Participants

A total of 405 participants took part in the study, aged 18 to 86 years (*M* = 48.64; *SD* = 16.41). Most participants (68.4%) reported receiving the recommended vaccines regularly, while 18% reported never receiving them and 13.6% reported receiving them only sometimes. For the annual influenza vaccine, 40.6% reported receiving it annually, 34.9% reported never receiving it, and 24.5% reported receiving it occasionally. Participants had to be 18 years or older and reside in Australia to be eligible for the study. No incentives were offered. (See Table 1 for demographic information). Almost half of the participants (*n* = 190; 47%) completed the follow-up survey.

**Table 1.** Demographic information (*N* = 405).

	N	%
Gender		
Male	115	28.4
Female	257	63.5
Other	13	3.2
Marital Status		
Married	180	46.9
Defacto	31	7.7
In a relationship	27	6.7
Separated/Divorced	53	13.1
Widowed	13	3.2
Never married	80	19.8
Children		
Yes	240	59.3
No	144	35.6
Employment		
Full-time	128	31.6
Part-time/casual	74	18.3
Study and work	24	5.9
Study only	31	7.7
Unemployed/home duties		
Annual Household Income		
$1–$20,766	32	7.9
$20,800–$103,999	185	45.7
≥ $104,999	114	28.1
Country of Birth		
Australia	272	67.2
United Kingdom	44	10.9
New Zealand	24	5.9
Other	38	9.4

### Materials

Demographic variables included gender, age, ethnicity, annual household income, education level, marital status, employment status, type of employment, occupation and parental status. Two attention-check questions were included in the survey to detect careless or inattentive responses.

#### Information source use and trust

Twelve information sources were identified based on existing literature: commercial television, commercial radio, Australian Broadcasting Corporation (ABC) news outlets (i.e., the main public broadcaster in Australia), the internet, social media, doctors, health professionals, politicians, state government, federal government, scientists/academics, and family/friends. These categories were selected based on prior COVID-19 vaccine and health communication literature (Chadwick et al., 2021; Demuyakor et al., 2021; Moujaess et al., 2021; Piltch-Loeb et al., 2021; Sallam, 2021). The Internet was defined as general online websites and search-based information sources, whereas social media was defined as interactive social networking platforms (e.g., Facebook, Instagram, Twitter/X). Similarly, doctors referred to “your doctor” reflecting general practitioners and medical doctors, whereas health professionals included other healthcare providers (e.g., nurses and allied health clinicians). Scientists/academics referred to scientific researchers and university-based experts. These categories were specified at a granular level to allow distinct information sources and messengers (e.g., government, health professionals, scientific experts, and media outlets) to be examined separately. Participants indicated the extent to which they used and trusted each information source on a 4-point Likert scale (0 = *none* to 3 = *a lot*), with higher scores indicating greater reliance on each source. This approach is consistent with prior COVID-19 research that has assessed vaccine perceptions and trust using single-item indicators of specific information sources and attitudes (Bertin et al., 2020; Osuji et al., 2022; Rosenthal & Cummings, 2021)

#### COVID-19 vaccination intention

Intention to receive a COVID-19 vaccine as soon as available was measured using four items developed in accordance with Ajzen’s (2006) guidelines. Participants responded on 7-point Likert scales (1 = *strongly disagree* to 7 = *strongly agree*). An example item was: “I intend to get a COVID-19 vaccine as soon as it is available to me”. Scores were averaged, with higher scores indicating greater intention to get the COVID-19 vaccine. The scale demonstrated excellent internal consistency (Cronbach’s α = .99).

#### COVID-19 vaccination uptake

COVID-19 vaccination uptake was assessed at follow-up using a single item: “Which of the following best describes your COVID-19 vaccination status?” Participants selected one of the following options: 1 = *I have received 2 vaccine doses*; 2 = *I have received 1 vaccine dose*; 3 = *I have an appointment to receive my 1st vaccine dose*; 4 = *I am not vaccinated yet, but I intend to be vaccinated*; 5 = *I am not vaccinated yet and I am not sure whether I will be vaccinated*; 6 = *I do not intend to be vaccinated*. Responses were recoded, with options 1 to 3 coded as 1 to indicate vaccination uptake or imminent uptake, and options 4 to 6 coded as 0 to indicate no vaccination uptake. Higher scores, therefore, indicate greater vaccination uptake. This recoding approach distinguishes behavioural uptake (including imminent uptake) from future intention or hesitancy, consistent with the staged nature of vaccine rollout at the time of data collection.

#### Data quality questions

Two questions were used to detect inattentive responding (e.g., please select option two to ensure you are paying attention; Maniaci & Rogge, 2014; Schroder et al., 2016). Participants (*n* = 146) who failed both questions were excluded prior to data analysis.

### Design and procedure

Ethical approval for the study was granted by the Human Research Ethics Committee of the University of the Sunshine Coast (approval number A211528), in accordance with the Declaration of Helsinki. The online survey was hosted using Qualtrics (https://www.qualtrics.com/au/). Data were collected between March and December 2021. March to June 2021 constituted Time 1, and October to December 2021 constituted Time 2. Time 1 data were collected during the early stages of the COVID-19 vaccine rollout in Australia, when vaccine availability was limited, and eligibility was restricted to priority groups. By contrast, Time 2 data were collected following broader vaccine availability and widespread public health messaging promoting vaccination uptake.

Participants were recruited at Time 1 through online channels, including paid Facebook advertisements, generic Australian community pages, and online forums. This recruitment strategy was selected to enable rapid recruitment of a geographically diverse Australian sample during the COVID‑19 pandemic. Recruitment materials invited Australian residents aged 18 years and older to participate in an online survey examining attitudes and beliefs about COVID-19 vaccines. After providing informed consent, participants completed the Time 1 survey, which took approximately 25 minutes. At the end of the survey, participants were asked whether they consented to being contacted for a brief follow-up survey. Responses for those who agreed were linked across time points using their provided email address. Participants who consented to follow-up were re-contacted approximately seven months later via email and invited to complete the Time 2 survey, which took approximately 2 minutes and assessed COVID-19 vaccination uptake.

### Data analysis

Data were analysed using IBM SPSS Statistics Version 31. Attrition analyses were conducted to compare participants who completed both survey waves with those who completed only Time 1. Independent-samples t-tests were used for continuous variables, and chi-square tests were used for categorical variables. Pearson correlation coefficients were calculated to examine relationships between COVID-19 information source use, trust in information sources, vaccination intention, and subsequent vaccination uptake. Paired-samples t-tests were conducted to examine discrepancies between the extent to which participants used COVID-19 vaccine information sources and the degree of trust placed in those sources. To examine the independent contribution of information source use and trust, multivariate analyses were also conducted. Multiple linear regression analyses were used to predict vaccination intention, and binary logistic regression analyses were used to predict vaccination uptake. Separate models were conducted for information source use and trust variables. All information-source variables were entered simultaneously in each model to account for overlap among sources and to identify independent predictors of vaccination intention and uptake. Statistical significance was based on α = .05.

## Results

### Attrition analysis

Attrition analyses comparing participants who completed both survey waves with those who completed Time 1 only are reported in the Supplementary Materials. Time 2 completers reported significantly higher baseline vaccination intentions and prior vaccine uptake but did not differ on key demographic variables, including age, education, employment, income, and non-English-speaking background.

### Use of COVID-19 information sources

Pearson correlation coefficients were calculated to examine the relationships between the use of COVID-19 vaccine information sources and participants’ vaccination intention at Time 1 and vaccination uptake at Time 2. At Time 1, use of information from State and Federal Governments, politicians, scientists/academics, health professionals, general practitioners, family and friends, ABC News outlets, newspapers, and commercial television was associated with stronger vaccination intention. In contrast, use of social media was associated with lower vaccination intention. Greater vaccination uptake was associated with the use of information from the State and Federal Governments, politicians, health professionals, general practitioners, ABC News outlets, newspapers, and commercial television at Time 2. In contrast, social media use was associated with lower vaccination uptake. No other correlations reached statistical significance (see Table 2).

**Table 2.** Pearson correlations between COVID-19 information sources, vaccination intention (*N* = 405), and vaccination uptake (*N* = 190).

Information Source	Intent to vaccinate [95% CI]	Vaccination uptake [95% CI]
Federal government	.35* [.26, .43]	.29* [.15, .41]
State government	.40* [.31, .48]	.38* [.26, .50]
Politicians	.28* [.18, .37]	.26* [.12, .39]
Scientists/academics	.15* [.05, .25]	.10 [−.04, .24]
Health professionals	.22* [.12, .31]	.15* [.01, .29]
General practitioner	.23* [.13, .32]	.24* [.10, .37]
Family and friends	.11* [.01, .21]	.09 [−.05, .23]
ABC News outlets	.40* [.32, .48]	.43* [.31, .54]
Newspapers	.11* [.01, .21]	.19* [.05, .32]
Commercial television	.10* [.00, .20]	.16* [.02, .30]
Commercial radio	.02 [−.08, .12]	.14 [.00, .28]
Internet	−.02 [−.12, .08]	−.03 [−.18, .11]
Social media	−.13* [−.23, −.03]	−.15* [−.29, −.01]

Note: Vaccination intention was measured at Time 1. Vaccination uptake was assessed at Time 2. *p < .05.

A multiple regression was conducted to examine whether the extent to which participants sourced COVID-19 vaccine information from different sources predicted vaccination intention. The overall model was significant, *F*(13, 361) = 11.66, *p* < .001, explaining 29.6% of the variance in intention. Greater use of ABC news outlets (β = .29, *p* < .001) and state government sources (β = .23, *p* = .003) was associated with higher intention. In contrast, greater use of commercial radio (β = −.12, *p* = .036) and social media (β = −.14, *p* = .007) was associated with lower intention. No other variables were significant predictors (see Supplementary Table 2).

A binary logistic regression was conducted to examine whether the extent to which participants sourced COVID-19 vaccine information from different sources predicted vaccine uptake behaviour. The overall model was statistically significant, χ^2^ (13) = 69.50, *p* < .001, explaining 53.7% of the variance in vaccination uptake. The Hosmer and Lemeshow test was non-significant, χ^2^ (8) = 7.32, *p* = .502, suggesting acceptable model fit. Greater sourcing of information from ABC news outlets, OR = 3.42, *p* < .001, and state government sources, OR = 6.51, *p* = .008, was associated with higher odds of vaccination uptake. In contrast, greater sourcing of information from social media was associated with lower odds of the behaviour, OR = 0.39, *p* = .021. No other variables were significant predictors (see Supplementary Table 4).

### Trust in COVID-19 information sources

Pearson correlation coefficients were calculated to examine the relationships between trust in COVID-19 vaccine information sources and vaccination intention at Time 1 and vaccination uptake at Time 2. At Time 1, greater trust in State and Federal Governments, politicians, scientists/academics, health professionals, general practitioners, family and friends, ABC News outlets, newspapers, and both commercial television and radio was associated with stronger vaccination intention, whereas trust in social media was associated with lower vaccination intention. Greater trust in State and Federal Governments, politicians, scientists/academics, health professionals, general practitioners, ABC News outlets, newspapers, and both commercial television and radio was associated with greater vaccination uptake at Time 2. In contrast, trust in both internet sources and social media was associated with lower uptake. No other correlations reached statistical significance (see Table 3).

**Table 3.** Pearson correlations between trust in COVID-19 information source, vaccination intention (*N* = 405), and vaccination uptake (*N* = 190).

Trust in Information Source	Intent to vaccinate [95% CI]	Vaccination uptake [95% CI]
Federal government	.54* [.47, .61]	.38* [.25, .50]
State government	.61* [.54, .67]	.45* [.33, .56]
Politicians	.43* [.35, .51]	.34* [.21, .46]
Scientists/academics	.43* [.35, .51]	.36* [.23, .48]
Health professionals	.58* [.51, .64]	.50* [.39, .60]
General practitioner	.65* [.59, .70]	.56* [.46, .65]
Family and friends	.18* [.08, .28]	.12 [−.02, .26]
ABC News outlets	.55* [.48, .61]	.46* [.33, .56]
Newspapers	.40* [.31, .48]	.31* [.17, .43]
Commercial television	.37* [.28, .45]	.30* [.17, .43]
Commercial radio	.29* [.20, .38]	.27* [.13, .40]
Internet	−.09 [−.19, .01]	−.17* [−.31, −.03]
Social media	−.17* [−.26, −.07]	−.29* [−.41, −.15]

Note: Vaccination intention was measured at Time 1. Vaccination uptake was assessed at Time 2. *p < .05.

A multiple regression was conducted to examine whether trust in COVID-19 vaccine information from different sources predicted vaccination intention. The overall model was significant, F(13, 369) = 35.07, *p* < .001, explaining 55.3% of the variance in intention. Greater trust in commercial television, β = .18, *p* = .022, ABC news outlets, β = .21, *p* < .001, one’s general practitioner, β = .21, *p* = .002, and state government sources, β = .22, *p* = .002 were associated with higher intention. Greater trust in the internet was associated with lower intention, β = −.10, *p* = .014. No other variables were significant predictors (see Supplementary Table 3).

A binary logistic regression was conducted to examine whether trust in COVID-19 vaccine information from different sources predicted vaccine behaviour. The overall model was statistically significant, χ^2^ (13) = 94.00, *p* < .001, explaining 67.6% of the variance in vaccination uptake. The Hosmer and Lemeshow test was non-significant, χ^2^ (8) = 2.32, *p* = .970, indicating good model fit. Greater trust in ABC news outlets OR = 2.72, *p* = .036, and state government sources, OR = 7.74, *p* = .045, was associated with higher odds of vaccination uptake. No other trust variables were significant predictors (see Supplementary Table 5).

### Differences between use of and trust in COVID-19 information sources

Paired-samples t-tests were conducted to examine differences between the extent to which participants accessed COVID-19 vaccine information from different sources and the level of trust placed in those sources. Significant discrepancies were observed between the use of information sources and trust. As shown in Table 4, trust in scientists and academics, health professionals, general practitioners, and family and friends was significantly higher than the frequency with which participants accessed information from these sources. Conversely, participants accessed social media and the internet more frequently than they trusted these sources.

**Table 4.** Paired samples t-tests (*N* = 190) between trust and COVID-19 information sources.

Baseline	*M* (*SD*) Information source	*M* (*SD*) Trust	*r*	*d*	*t*	*p*
Federal Government	1.09 (.95)	1.05 (10)	.621***	.04	.601	.548
State Government	1.18 (.96)	1.21 (.10)	.682***	−.04	−.556	.579
Politicians	.74 (.80)	.71 (.77)	.600***	.05	.625	.533
Scientists/academics	2.37 (.76)	2.58 (.70)	.447***	−.27	−3.693	<.001
Health professionals	1.67 (1.14)	2.34 (.90)	.458***	−.62	−8.546	<.001
General Practitioner	1.45 (1.16)	2.32 (.94)	.484***	−.80	−10.970	<.001
Family and friends	1.11 (.84)	1.30 (.86)	.695***	−.29	−3.937	<.001
ABC News outlets	1.61 (1.10)	1.57 (1.05)	.776***	.06	.816	.416
Newspapers	.89 (.99)	.87 (.85)	.626***	.03	.450	.653
Commercial television	.55 (.86)	.61 (.73)	.577***	−.09	−1.189	.236
Commercial radio	.31 (.63)	.55 (.69)	.528***	−.38	−5.150	<.001
Internet	2.01 (.86)	1.30 (.78)	.495***	.86	11.874	<.001
Social media	1.08 (.94)	.66 (.78)	.613***	.55	7.519	<.001

Note: *<.05; **<.01; ***<.001.

## Discussion

This study aimed to examine the role of information source use and trust during the COVID‑19 pandemic in relation to vaccination intention and uptake. In line with the hypotheses, both engagement with and trust in the state government and ABC News predicted higher vaccination intention and uptake. In contrast, reliance on social media and internet sources was associated with lower vaccination outcomes. The findings also revealed a discrepancy between trust and use: participants reported greater trust in professional and institutional sources, such as doctors, health professionals, scientists, and academics, yet more frequently relied on social media and internet-based platforms for COVID-19 vaccine information. Together, these findings suggest that vaccination behaviour may be shaped not only by the perceived credibility of information sources but also by the characteristics and accessibility of the information environments in which individuals engage.

The findings highlight the importance of information environments during the COVID-19 pandemic. At the bivariate level, use and trust in a broad range of sources, including governments, scientists, health professionals, and mainstream media, were positively associated with both vaccination outcomes. These findings are consistent with previous research demonstrating that exposure to credible information and trust in public health and institutional sources are associated with greater vaccine acceptance (Geller et al., 2025; Hassan et al., 2021; Jennings et al., 2021; Liu et al., 2023; Thaker et al., 2023). However, once overlap among sources was accounted for, a more selective pattern emerged. Across both the source-use and trust models, ABC News and state government sources were the only sources that independently predicted both vaccination intention and uptake. This suggests that vaccination outcomes were not simply associated with accessing more information or trusting more sources. Rather, certain sources may be uniquely positioned to predict both vaccination intentions and behaviour.

One explanation for the consistent role of state government sources is that these sources played a central role in implementing the vaccine rollout within their jurisdictions, including coordinating vaccination services, booking systems, clinic delivery, and region-specific public health advice (Australian Government, 2021; Choiseul et al., 2021). This interpretation is consistent with implementation and behavioural science research demonstrating that vaccination uptake is influenced not only by attitudes and beliefs, but also by access, convenience, and the organization of delivery systems, which facilitate the translation of intention into behaviour (e.g., Davies et al., 2026; Pilar et al., 2022). Information from state governments may therefore have supported both motivation to vaccinate, and navigation of the practical steps required to act on that intention. Similarly, ABC News operates under a public service model that emphasises accurate and impartial reporting in line with recognised standards of objective journalism (Australian Broadcasting Corporation, 2023). This may have enhanced its credibility during a period of uncertainty and rapidly evolving public health advice. Individuals who relied on and trusted these sources may therefore have been more likely to receive both accurate information regarding vaccination and clear guidance on how to access vaccination services. Together, these findings suggest that information sources are particularly influential when they combine credibility with practical and actionable guidance, thereby supporting the translation of vaccination intentions into behaviour.

The findings also showed that trust in some sources was associated with vaccination intention but not uptake. Specifically, commercial radio predicted lower vaccination intention, while trust in general practitioners and commercial television predicted higher intention only. Commercial radio often incorporates opinion-based and discussion-driven formats that may shape perceptions and intention without necessarily influencing subsequent behaviour, particularly when messaging emphasises uncertainty or contested information (Burke et al., 2021; Ruggeri et al., 2024; Williamson & Tarfa, 2022). Conversely, communication-based interventions can improve knowledge and intention, but are less consistently effective at changing behaviour (Li et al., 2022). This pattern is well documented in the intention–behaviour gap, whereby intention is a key predictor of behaviour but does not always result in action (Ajzen, 1991; Sheeran & Webb, 2016; Webb & Sheeran, 2006).

Findings relating to online and social media environments were likewise nuanced. In alignment with previous research (Allington et al., 2023; Liu et al., 2023; Loomba et al., 2021), social media use predicted both lower vaccination intention and uptake. Features of social media environments, including algorithmic curation, misinformation exposure, emotionally engaging content, and the reinforcement of like-minded viewpoints, have been consistently linked to increased vaccine hesitancy and reduced trust in institutional health messaging (e.g., Allington et al., 2023; Loomba et al., 2021; Ruggeri et al., 2024). In contrast, only trust in the internet independently predicted lower vaccination intention. This may reflect the breadth and variability of online information environments, which contain information of highly variable quality, including misinformation and conflicting claims. Previous research has demonstrated that exposure to online vaccine misinformation can reduce vaccination intentions and increase hesitancy (e.g., Loomba et al., 2021; Zimmerman et al., 2023). As such, greater trust in internet-based information may increase susceptibility to uncertain or misleading content, potentially undermining vaccination intentions. However, given the correlational nature of the present study, alternative explanations such as selective exposure and reverse causality cannot be ruled out.

An additional finding was the discrepancy between trusted and frequently used information sources. Although participants reported high trust in doctors, health professionals, scientists, academics, and family and friends, they more often relied on social media and internet sources for COVID-19 vaccine information. Similar trust-use gaps have been documented previously (Jennings et al., 2021; Tayal & Bharathi, 2021), highlighting an ongoing challenge for public health communication. This discrepancy is important because reliance on less-trusted sources does not necessarily imply endorsement of their credibility; it may instead reflect pragmatic factors such as time constraints, information overload, platform familiarity, and accessibility. The design and affordances of online platforms, including ease of access and emotionally engaging content, can further reinforce reliance on these sources (e.g., Schreiner et al., 2021), even when users report lower trust in them. Addressing this gap requires improving the accessibility and user experience of reputable sources, integrating credible content within commonly used platforms, and encouraging proactive planning to seek out reliable information. These approaches are likely to remain pertinent for future vaccination campaigns and other large-scale health initiatives.

Several limitations should be considered when interpreting these findings. The sample included an overrepresentation of White, female, and higher-income participants, which may limit the generalisability of the findings. Research indicates that higher income is associated with greater acceptance of vaccination, potentially due to better access to healthcare, higher education, and greater health literacy (Bono et al., 2021; Dash et al., 2021; Reno et al., 2021). Individuals who regularly engage with online platforms may differ from the broader population in both their exposure to and reliance on internet-based and social media information sources. Consequently, the prevalence of online information use observed in the present study may not fully reflect patterns of information seeking in the wider Australian population. In addition, although attrition between Time 1 and Time 2 was moderate and unrelated to key demographic characteristics, participants who completed follow-up reported higher baseline vaccination intentions and prior vaccine uptake behaviours. This selective attrition may limit the representativeness of the follow-up sample and should be considered when interpreting findings relating to vaccination uptake. Together, the demographic and recruitment profile may have biased the findings towards higher baseline vaccination intentions and uptake, potentially overestimating the strength of associations observed for trusted institutional and professional information sources.

A second limitation concerns the longitudinal design and the interpretation of associations. Although vaccination uptake was assessed at a later time point than information source use and trust, the relationship between information source use, trust, and vaccination intention was measured concurrently, precluding conclusions about directionality. Consequently, it remains plausible that individuals selectively engaged with information sources consistent with their existing intentions, rather than source use shaping intention. While the temporal separation between baseline predictors and subsequent vaccination uptake provides somewhat stronger evidence for a directional relationship, these associations may still be confounded by baseline vaccination intentions or other unmeasured factors that influenced both information-seeking behaviour and later vaccination decisions. Future research using longitudinal cross-lagged designs, experimental approaches, or interventions targeting information source exposure and trust would help clarify the direction and causal status of these relationships.

The findings have several implications for public health communication. The results suggest that vaccination campaigns may benefit from working through trusted, widely used information channels while ensuring that accurate information remains accessible in online environments. The observed trust–use discrepancy further highlights the importance of not only maintaining public trust in health institutions, but also ensuring that credible information is available through the platforms people routinely use. These considerations may be relevant to future vaccination campaigns and other public health emergencies in which information quality and trust play important roles in shaping behaviour.

## Conclusion

In conclusion, this study demonstrates that the use of information sources and trust play meaningful roles in shaping both COVID‑19 vaccination intention and uptake. Although a wide range of information sources were associated with vaccination outcomes, state government and ABC News sources emerged as the most consistent predictors across both information-use and trust models. In contrast, greater reliance on social media and trust in internet-based information were associated with less favourable vaccination outcomes. The findings also revealed a discrepancy between trusted and frequently used information sources, suggesting that factors beyond perceived credibility alone influence information-seeking behaviour. Together, these findings demonstrate the importance of considering both trust and information environments when seeking to understand and support vaccination behaviour during public health emergencies.

## Data Availability

Data, study code, analysis, and output are available on OSF: *Have You Done Your Research? How Information Sources and Trust Shape COVID-19 Vaccine Intentions and Uptake* https://osf.io/kse9p/?view_only=7d39f685f3c94cc88f3c5119e1b52fe5

## Open scholarship

This article has earned the Center for Open Science badges for Open Data and Open Materials through Open Practices Disclosure. The data and materials are openly accessible at https://doi.org/10.17605/OSF.IO/KSE9P
